# Reddit Perspectives on Tonsillectomy Procedure: Sentiment and Thematic Analysis

**DOI:** 10.7759/cureus.74254

**Published:** 2024-11-22

**Authors:** Ava Herzog, Victoria Vought, Rita Vought, Andrew Lee, Rahul Sharma, Brian Manzi

**Affiliations:** 1 Department of Otolaryngology, Albany Medical College, Albany, USA; 2 Department of Otolaryngology, Rutgers University New Jersey Medical School, Newark, USA

**Keywords:** post-operative pain management, reddit, sentiment analysis, social media analytics, tonsillectomy

## Abstract

Introduction: Every year, 530,000 tonsillectomies are performed in the United States. Many patients use social media for medical advice and support. This study investigates Reddit perspectives to identify the current needs of tonsillectomy patients.

Methods: The “Top” 500 posts of the r/tonsillectomy subreddit community (3600 members) were qualitatively classified by theme, author, and post content. A Python sentiment analysis package, Valence Aware Dictionary sEntiment Reasoner (VADER), assigned summary Compound scores (-1=most negative, +1=most positive). A Lexicon-based *syuzhet* package analyzed the emotions of each post. Word frequency analysis elucidated top descriptors. Kruskal-Wallis and Mann-Whitney tests were used to determine statistical significance.

Results: Posts were themed as Attitudes/Experiences (n=440, 88%), Medical/Procedural (n=9, 1.8%), or Both (n=51, 10.2%). Most were patient-authored (n=474, 94.8%), with many sharing personal anecdotes (n=476, 95.2%) and seeking validation (n=109, 21.8%). Few involved preoperative (n=15, 3%) or postoperative (n=45, 9%) concerns, with no operative or medication-related questions. VADER analysis revealed an average Compound score of 0.26, with 320 Positive, 179 Negative, and one Neutral post. Fear and trust had the most associated words from the National Research Council (NRC) Emotion dictionary. Common descriptors were “pain” (n=2063), “throat” (n=718), “water” (n=675), “feel” (n=576), and “ice” (n=328).

Conclusion: Most Reddit users viewed their tonsillectomy experience as positive. Many shared experiences and a few of them involved clinical questions. The descriptor “pain” was particularly prevalent. Providers should supply detailed pain-related expectations. Reddit may allow surgeons to better grasp how real patients manage pain, combining home remedies with prescribed medications. Care can be enhanced through personalized support that aims to minimize fear and improve patient-physician trust.

## Introduction

Tonsillectomies are one of the most common pediatric surgeries, with over 530,000 procedures performed annually in the United States [1]. In 2006, 40.3% of tonsillectomies in the United States were performed in adults [2]. Tonsillectomies are indicated for children with obstructive sleep apnea, recurrent tonsil infections, antibiotic allergies, or peritonsillar abscesses [3]. In adults, they are also used to treat obstructive sleep apnea syndrome, sleep disorders, suspected malignancies, or infections [4]. Candidates for tonsillectomy face a significant surgical decision, as there are postoperative risks of bleeding, dehydration, and respiratory distress [5]. In addition, a minority of children with obstructive sleep apnea can resolve their symptoms without a tonsillectomy, eliciting some ambiguity among parents as to how beneficial the surgery will be for their children [6]. Parents may seek information from the internet when making this decision, highlighting the importance of social media for medical advice.

Patients often use social media platforms such as Twitter or Facebook to gather real-time medical information and advice [7]. Reddit, an anonymous social media site, boasts a large community and allows users to post content to which other users can post a reply. Unlike most platforms, Reddit contains topic-specific forums called “subreddits” that consist of members with a common interest. Medical subreddits have been used in past studies to assess patient perspectives on prevalent medical topics [8]. Reddit has become increasingly popular outside of the United States in recent years, including its use as a data source in research [9].

Sentiment analysis can produce objective quantifiable measures of patient perspectives. Valence Aware Dictionary and sEntiment Reasoner (VADER) is a rule-based sentiment Python package that determines written text sentiment and its intensity through a Compound score [10]. This tool has been applied in previous social media analyses of glaucoma care [11]. The National Research Council (NRC) Emotion Lexicon-based natural language processing *syuzhet* package scores the written text for eight different emotions (anger, fear, anticipation, trust, surprise, sadness, joy, and disgust) and two sentiments (positive or negative) [12, 13]. Content can contain more than one emotion, allowing for a more complex analysis. VADER and *syuzhet* tools have been previously utilized together to analyze Italian Twitter users’ sentiments and emotions in their tweets [14]. These can be used in conjunction with word frequency analysis to determine commonly mentioned words or topics within a dataset [15]. Therefore, the objective of this study is to apply VADER, *syuzhet*, and word frequency analyses of Reddit tonsillectomy-related content as comprehensive tools to investigate how social media platforms are used to discuss tonsillectomies. Posts are expected to display mixed sentiments of patients anticipating or recovering from the tonsillectomy procedure, with subreddit users providing support, giving advice, offering reassurance, and detailing personal experiences.

## Materials and methods

Member posts and comments were abstracted from the r/tonsillectomy community (approximately 3600 members) of Reddit (reddit.com) using a Reddit application programming interface (API). Our criteria utilized the filters “Top” for post popularity and “All Time” for time frame to identify 500 posts for inclusion. These filters were chosen to obtain posts with the highest upvotes and engagement since the creation of the r/tonsillectomy subreddit, likely resonating with many subreddit users. After examining the content of the posts, 500 posts were considered by our team necessary to achieve thematic saturation.

The posts from Reddit users between July 2021 and November 2023 were considered in our analysis. The title of each post and “Best” (proxy for popularity) two comments under each post were also recorded, along with author information, post score, and upvote ratio. Each post can be upvoted or downvoted by other community members to promote or demote the post on the community page. The post score represents the net number of votes, where each upvote adds one point (+1) and each downvote subtracts one point (-1). The upvote ratio represents the percentage of all votes (upvotes and downvotes) that were upvotes.

Using the title and contents, posts were classified by self-reported author type (tonsillectomy patient, parent/caregiver of a tonsillectomy patient, medical professional, other/unknown). Posts and comments were also classified by theme: Medical/Procedural, Attitudes/Experiences, or Both. Procedural categories included pre-operational, operative, postoperative, and medication-related content. Attitudes and experiences included posts that sought validation for symptoms and experiences or posts that focused on sharing an anecdote.

Reddit posts were then analyzed for sentiment using VADER to generate a Compound score. The VADER Compound score is a rating system that ranges from -1 to +1, with -1 as the most negative sentiment and +1 as the most positive sentiment. The score assigned to each post represents the proportion of how much of that sentiment exists. The Negative, Positive, and Neutral scores for each post equal one. VADER also uses the Compound score to categorize posts as Negative, Neutral, or Positive.

Word frequency analysis was applied to posts and comments as well. The top five words and bigrams overall, as well as the top five words and bigrams in the Most Positive (Compound score > 0.75) and Most Negative posts (Compound score <0) were recorded [16, 17]. Bigrams are pairs of consecutive words within text, such as “my throat” or “to eat.” The most used descriptor when stratified by qualitative category and theme for both Most Positive and Most Negative posts was also recorded. Non-relevant words such as “am,” “and,” “but,” and “I” were excluded from single-word frequency analysis.

Post sentiment was further analyzed by the R library package *syuzhet,* which applies an emotion dictionary to score posts with VADER-identified negative or positive sentiment (excludes neutral posts) for the following eight emotions: anger, fear, anticipation, trust, surprise, sadness, joy, and disgust. Scores are calculated for each emotion using the NRC algorithm, with a post containing three words in the “sadness” word list scored as a three in the “sadness” emotion category. To obtain scores representing emotion sentiment per theme, the number of unique words found for each emotion via the emotion dictionary was recorded along with the top three most frequent words for each theme. Kruskal-Wallis and Mann-Whitney H tests were utilized to assess statistical significance with an alpha value of 0.05.

Ethics approval

This study adhered to the Declaration of Helsinki, and no Institutional Review Board approval was required. Regardless of Reddit’s public availability, it is important to review ethical considerations when utilizing social media data.

## Results

This study evaluated 500 Reddit posts. The majority (94.8%) of the posts were authored by tonsillectomy patients and 5.2% were written by other or unknown users (Table 1). No posts were authored by medical professionals or parents/caregivers of tonsillectomy patients. The posts’ scores (net upvote/net downvote) ranged from 6 to 84 (average of 12.22), with a mean upvote ratio of 0.96. As many as 210 posts had an upvote ratio of 0.8-0.99 and 290 posts had an upvote ratio of 1, indicating that posts were well received by other Reddit users.

**Table 1:** Author Type and Topic Coverage of Reddit Posts Evaluated.

Author Type	Number of Posts (%)	Representative Post Excerpt
Parent/caregiver of tonsillectomy patient	0 (0%)	-
Tonsillectomy patient	474 (94.8%)	“i had my surgery on march 29…”
Medical professional	0 (0%)	-
Other/do not know	26 (5.2%)	“I am 32, considering getting a tonsillectomy…”
Qualitative Analysis		
Preoperative question	15 (3%)	“Will swollen lymph nodes go down? Hello all, I (19F) have my surgery scheduled next week…”
Operative question	0 (0%)	-
Postoperative question	45 (9%)	“When can you have sex again after the surgery?”
Medication-related question	0 (0%)	-
Seeking validation for symptoms/experiences	109 (21.8%)	“I had the most discomfort for the first two days post op but now i could honestly chew and eat with ease. is this normal?”
Sharing anecdote	476 (95.2%)	“I am 5 weeks post op this week and this community was a huge help and was pretty much the only thing I was reading during my recovery - I have a few things to share that hopefully will help a few of you also going through this!”
Categorized Themes		
Medical/Procedural	9 (1.8%)	“What are some must have items I need to have on hand during recovery? Ice pack? Heat packs?”
Attitudes/Experiences	440 (88%)	“Finally… FINALLY… a glimpse of normal… y’all… i’m so happy i’ve cried all day”
Both	51 (10.2%)	“I’m anxious. But I need to get this done. ENT said my tonsils are approximately five times larger than they should be. I’m chronically sick. Wake up every day with sore throats and regurgitate tonsil stones regularly.”

A qualitative analysis of procedural topics showed that 9% of posts included a postoperative question, 3% a preoperative question, and no posts contained operative or medication-related questions. A discussion of attitudes and experiences was prevalent, with 95.2% of posts sharing an anecdote and 21.8% seeking validation for symptoms or experiences. Due to the multifaceted nature of the content of the posts, they could be classified under multiple categories for qualitative analyses (i.e. preoperative question and sharing an anecdote). Posts were themed as Attitudes/Experiences (88%), Medical/Procedural (1.8%), or Both (10.2%) (Table 1).

VADER analysis produced an average Negative, Neutral, Positive, and Compound score of sentiment per Reddit post (including title and comments). These scores were 0.11, 0.75, 0.14, and 0.26, respectively (Table 2). VADER also classified overall Compound ratings as Negative (35.8%), Neutral (0.2%), or Positive (64%) (Table 3).

**Table 2:** VADER Sentiment Scoring of Reddit Posts. Scores represent the proportion of text that expresses a particular sentiment. Negative, Neutral, and Positive scores range from 0 to 1. Compound scores combine the negative, neutral, and positive scores, ranging from -1 being most negative and +1 being most positive. Abbreviation: VADER=Valence Aware Dictionary sEntiment Reasoner

VADER Score Per Reddit Post (incl. title and comments)	Average Score
Negative score	0.11
Neutral score	0.75
Positive score	0.14
Compound score	0.26

**Table 3:** VADER Sentiment Rating of Reddit Posts. Abbreviation: VADER=Valence Aware Dictionary sEntiment Reasoner

VADER Ratings by Reddit Post (incl. title and comments)	Number of Posts (%)
Negative	179 (35.8%)
Neutral	1 (0.2%)
Positive	320 (64%)

The analysis of VADER Compound scores showed no differences in the sentiment of the post based on author type (p=0.64). A comparison by topic coverage revealed statistically different Compound scores (p=0.0031), with anecdotal posts having more Positive scores (0.27) than posts with a preoperative question (0.020), postoperative question (-0.024), or seeking validation for symptoms or experiences (-0.032). A similar significant finding (p=0.014) was obtained between post themes, with average Compound scores for Attitudes/Experiences, Medical/Procedural, and Both as 0.30, 0.06, and -0.01, respectively (Table 4).

**Table 4:** Average Sentiment Score of Reddit Posts by Author Type, Qualitative Analysis, and Theme. Kruskal-Wallis and Mann-Whitney tests (α = 0.05). Abbreviation: VADER=Valence Aware Dictionary sEntiment Reasoner

			P-Value
Author Type		VADER Score	0.64
Parent/caregiver of tonsillectomy patient			
	Mean ± SD	n/a	
	Median (Range)	n/a	
Tonsillectomy patient			
	Mean ± SD	0.27 ± 0.85	
	Median (Range)	0.84 (-1.00 – 1.00)	
Medical professional			
	Mean ± SD	n/a	
	Median (Range)	n/a	
Other/unknown			
	Mean ± SD	0.17 ± 0.90	
	Median (Range)	0.79 (-0.99 – 0.99)	
Qualitative Analysis			0.0031
Preoperative question			
	Mean ± SD	0.02 ± 0.91	
	Median (Range)	-0.31 (-1.00 – 0.99)	
Operative question			
	Mean ± SD	n/a	
	Median (Range)	n/a	
Postoperative question			
	Mean ± SD	-0.02 ± 0.85	
	Median (Range)	-0.38 (-1.00 – 0.99)	
Medication-related question			
	Mean ± SD	n/a	
	Median (Range)	n/a	
Seeking validation for symptoms/experiences			
	Mean ± SD	-0.03 ± 0.87	
	Median (Range)	-0.38 (-1.00 – 1.00)	
Sharing anecdote			
	Mean ± SD	0.27 ± 0.85	
	Median (Range)	0.86 (-1.00 – 1.00)	
Categorized Themes			0.01
Medical/Procedural			
	Mean ± SD	0.06 ± 0.79	
	Median (Range)	-0.31 (-0.93 – 0.99)	
Attitudes/Experiences			
	Mean ± SD	0.30 ± 0.84	
	Median (Range)	0.88 (-1.00 – 1.00)	
Both			
	Mean ± SD	-0.01 ± 0.88	
	Median (Range)	-0.03 (-1.00 – 0.99)	

Word frequency analysis identified the most used words and phrases among Reddit posts (Table 5). There were a total of 199,557 words and 8,355 unique words in the posts. Single-word analysis of the entire dataset identified “pain” (n =2063), “throat” (n = 718), “water” (n = 675), “feel” (n = 576), and “ice” (n = 328) as the most used relevant words. Positive Reddit posts frequently mentioned “pain” (n=775), “surgery” (n=518), “water” (n=365), “recovery” (n=362), and “good” (n=227), while negative Reddit posts most frequently contained “pain” (n=1165), “surgery” (n=328), “water” (n=275), “eat” (n=240), and “ice” (n=210).

**Table 5:** Single-Word and Bigram Frequency Analysis of Reddit Posts by Sentiment, with Most Positive and Negative Reddit Posts by Category.

Category of Reddit Posts	Single-Word Analysis		Bigram Analysis	
	Word	Frequency	Word	Frequency
Overall				
	pain	2063	the pain	447
	throat	718	my throat	329
	water	675	I can	278
	feel	576	to eat	253
	ice	557	post op	234
Positive				
	pain	775	the pain	184
	surgery	518	you can	129
	water	365	to eat	114
	recovery	362	I feel	85
	good	227	my recovery	70
Most Positive				
Preoperative question	surgery	14	my tonsils	7
Postoperative question	pain	31	my throat	10
Seeking validation	pain	52	my throat	17
Sharing anecdote	pain	768	the pain	183
Negative				
	pain	1165	the pain	234
	surgery	328	pain meds	106
	water	275	the worst	93
	eat	240	I feel	80
	ice	210	ear pain	55
Most Negative				
Preoperative question	stones	25	tonsil stones	14
Postoperative question	pain	70	the pain	23
Seeking validation	pain	154	the pain	38
Sharing anecdote	pain	1157	the pain	233

Of all the Reddit posts, bigram analysis identified the following top descriptors: “the pain” (n = 447), “my throat” (n = 329), “I can” (n = 278), “to eat” (n = 253), and “post op” (n = 234). Analysis of the Most Positive and Most Negative posts revealed respective top five bigram phrases of “the pain,” “you can", “to eat,” “I feel,” “my recovery” versus “the pain,” “pain meds", “the worst,” “I feel,” and “ear pain.”

When grouping posts by qualitative category, “pain” was the most used descriptor in Most Positive and Most Negative posts for postoperative questions, seeking validation for symptoms/experiences, and sharing anecdote categories (Table 5). “The pain” or “my throat” were the top bigrams for those categories. Preoperative question posts had “surgery” (n=14) and “my tonsils” (n=7) as most prevalent in Most Positive posts, and “stones” (n=25) and “tonsil stones” (n=14) in Most Negative posts.

Inclusion criteria identified 499 posts for *syuzhet* emotion analysis. The number of unique associated words linked to an emotion of the Lexicon Emotion Dictionary helps quantify how diverse the vocabulary is for each emotion. The three emotions with the most associated unique words for Medical/Procedural posts were trust (n=24), fear (n=24), and anticipation (n=23) (Table 6). Of the Attitudes/Experiences posts, fear (n=291), trust (n=291), and sadness (n=258) were the most prevalent. Themed Both posts’ top emotions were fear (n=102), trust (n=101), and sadness (n=96) (Table 7). “Pain,” “feeling,” “good,” “bad,” “surgery,” “painful,” “bleeding,” “food,” and “pretty” were descriptors found to be associated with more than one emotion.

**Table 6:** Number of Lexicon Emotion Dictionary-Associated Words of Themed Reddit Posts. The number of unique associated words linked to an emotion of the Lexicon Emotion Dictionary quantifies how diverse the vocabulary is for each emotion.

Emotion	Medical/Procedural	Attitudes/Experiences	Both
Anger	13	218	79
Anticipation	23	233	82
Disgust	11	201	72
Fear	24	296	102
Joy	14	200	63
Sadness	19	258	96
Surprise	10	132	43
Trust	24	291	101

**Table 7:** Most Frequent Single Words Associated with Lexicon Emotion Dictionary of Themed Reddit Posts.

Emotion	Medical/Procedural	Attitudes/Experiences	Both
Anger	Bad (n=3)	Bad (n=319)	Bad (n=30)
	Feeling (n=2)	Feeling (n=272)	Painful (n=15)
	Painful (n=2)	Sore (n=159)	Sore (n=13)
Anticipation	Good (n=5)	Time (n=403)	Time (n=38)
	Long (n=4)	Good (n=347)	Good (n=25)
	Pretty (n=3)	Feeling (n=272)	Thought (n=17)
Disgust	Bad (n=3)	Bad (n=319)	Bleeding (n=33)
	Feeling (n=2)	Feeling (n=272)	Bad (n=30)
	Painful (n=2)	Bleeding (n=228)	Painful (n=15)
Fear	Painful (n=10)	Pain (n=1925)	Pain (n=139)
	Surgery (n=6)	Surgery (n=822)	Surgery (n=81)
	Bad (n=3)	Bad (n=319)	Bleeding (n=33)
Joy	Food (n=6)	Good (n=347)	Good (n=25)
	Good (n=5)	Food (n=283)	Food (n=18)
	Pretty (n=3)	Feeling (n=272)	Pretty (n=16)
Sadness	Pain (n=10)	Pain (n=1925)	Pain (n=139)
	Surgery (n=6)	Surgery (n=822)	Surgery (n=81)
	Bad (n=3)	Bad (n=319)	Bleeding (n=33)
Surprise	Good (n=5)	Good (n=347)	Good (n=25)
	Mouth (n=3)	Feeling (n=272)	Hope (n=13)
	Deal (n=2)	Mouth (n=221)	Feeling (n=10)
Trust	Food (n=6)	Don't (n=369)	Don't (n=44)
	Good (n=5)	Good (n=347)	Good (n=25)
	Pretty (n=3)	Food (n=283)	Good (n=18)

## Discussion

Active patient participation online can positively promote health outcomes, boost patient satisfaction, and improve efficiency and quality of healthcare [18]. Since many patients rely on social media to select physicians and treatment plans, providers who discuss online educational materials with patients tend to have higher satisfaction rates. This suggests that understanding and validating patients’ use of social media can help establish strong patient-physician bonds and address patients’ multifaceted needs [19]. Social media engagement likely influences patient outcomes, warranting a thorough examination of its impact. In this study, we evaluated the discussion of the tonsillectomy procedure on a popular platform, Reddit, using VADER, word frequency, and *syuzhet* analyses.

Analysis of the post content

A qualitative analysis of posts revealed that 95.2% of posts shared anecdotes and 21.8% sought validation for symptoms/experiences, while only 12% of posts involved clinical questions (Table 1). Few posts involved postoperative (9%) or preoperative (3%) concerns, with none incorporating operative or medication-related questions. This percentage of clinical concerns is lower than the approximate 20% postoperative complication rate of tonsillectomies [20]. Our study corroborated previous findings with anxiety and generalized pain as the most reported symptoms on social media sites [21], with 21.8% of posts seeking validation and “pain” (n=2063) being the top descriptor in Overall posts.

VADER analysis

Sentiment analysis revealed a positive average Compound VADER score (Table 2). When stratified by qualitative category (Table 4), posts sharing experiences were the Most Positive, suggesting that users had high success rates. They may also have felt a sense of community within the r/tonsillectomy subreddit. Tonsillectomy patients are expected to have improved quality of life postoperatively, with less frequent episodes of sore throat pain, fewer doctor visits, and reduced reliance on analgesic or antibiotic medications [22]. A 2016 comparative analysis indicated that 97.2% of adult tonsillectomy patients were considered cured [23]. Thus, tonsillectomies produce generally favorable outcomes, with patients depicting overall positive experiences on Reddit.

Word frequency analysis

An analysis of word frequency also identified subjects important to tonsillectomy patients. When examining Overall, Positive, and Negative word frequency, “pain” and “the pain” were the most frequent single word and bigram used, respectively (Table 5). “Pain” was also the most common word in Most Negative and Most Positive posts that asked a postoperative question, sought validation, or shared an anecdote (Table 5). Pain was described by users as severe earaches, headaches, and discomfort when speaking, chewing, swallowing, and sleeping. More specifically, pain was denoted by the following: “burning ear pain that felt like pins in my ears,” “throat was on fire,” “chewing felt like someone was stabbing me in my throat,” “super bad strep throat,” and “tongue was still a small/medium balloon.” Patients self-reported prescriptions of acetaminophen, ibuprofen, tapentadol, oxycodene, naproxen, paracetamol, vicodin, panadol osteo, as well as anti-inflammatory medications and steroids for swelling. Home remedies, alternative pain management strategies, and recovery tips were also discussed. Patients noted better sleep with ice pack head wraps, neck pillows, mouth tape, humidifiers, and directly icing the neck. Hot soup, tea with honey, ice chips, and chewing gum were commonly shared as the best foods to ease pain. Users recommended having anti-nausea pills, stool softeners, and laxatives on hand to prepare for pain medication side effects. In general, patients experienced the most severe pain during days one and two post-operatively, with near full recovery at two weeks.

Literature has indicated that there is no clear consensus on the most effective pain management regimen following tonsillectomies, with providers often undertreating pain [24]. This is especially true in the pediatric population to avoid narcotics or other strong pain medications [25]. Research has indicated that patients or parents may not be fully aware of how to best manage postoperative pain at home, emphasizing the need for detailed instruction and education. Presenting specific pain management approaches, such as Q4H (every four hours) acetaminophen dosing and Q8H (every eight hours) ibuprofen dosing, and instructing parents to log pain medication doses given may improve these concerns [24]. Increased focus on pain management may improve patient satisfaction, as other studies have also identified pain management as influential in physician reviews [17].

Terms related to eating and drinking, such as “eat,” “ice,” “water,” and “to eat,” were also extensive among Reddit posts (Table 5). A 2016 systematic review on post-tonsillectomy dietary advice concluded that there is much discrepancy among physician suggestions [26]. The lack of consistency among diet postoperative routines may cause patients to be concerned about their path toward recovery, turning to online forums to seek validation. Clearer postoperative instructions that outline what patients should and should not do after a tonsillectomy may mitigate these feelings of uncertainty and confusion.

*Syuzhet* analysis

Fear and trust had the most unique associated words, indicating a more varied or complex expression of these emotions among Reddit users (Table 6). Similar to our sentiment analysis findings, “pain” was the most frequent word used in association with fear and sadness in Attitudes/Experiences posts (Table 7). These emotions in a surgical setting can be attributed to uncertainty, concerns about complications and pain, and loss of control. In a clinical setting, inaccurate expectations and low levels of trust in physicians have been associated with poorer patient-physician relationships. Preoperative anxiety among surgical patients can be reduced by providing specific procedural information to patients [27]. In addition to our emotion-related findings, a 2003 prospective study concluded that 17% of pediatric tonsillectomy patients developed depression symptoms three weeks postoperatively [28]. Increased psychological preparation and depression screening have been indicated as potential ways to mitigate this high prevalence [29]. Fear of a surgical procedure is a natural response often reported by patients. Despite the prevalence of fear and trust within our dataset, patients consistently shared positive experiences, likely because of successful surgical outcomes, supportive care, and an improved quality of life.

Study limitations

There are a few limitations to our study. First, the dataset was obtained by filtering for “Top” Reddit posts, disregarding posts with low upvote ratios, such as specific medical questions or concerns, and potentially introducing selection bias. Additionally, Reddit’s underlying dynamics may have impacted results, with Reddit’s voting system influencing which posts are visible to users. Reddit requires users to have an established account to post or comment, further limiting the user types within the dataset. With most posts suspected to be authored by adults, our findings may not fully reflect concerns across all age groups. Individuals with prosocial tendencies or unhappy feelings are more likely to post on social media than individuals with positive attitudes, constricting the scope of patient personalities expressed on Reddit [30]. Since personal anecdotes are subjective, post content may have been misinterpreted by our team - an inherent limitation of sentiment analyses.

Studying Reddit posts contextualizes what providers already know from the clinic. This data offers first-hand accounts of diverse patient experiences. While this data cannot be generalized to the entire tonsillectomy patient population, it provides valuable qualitative insight that can be used alongside clinical knowledge to best optimize patient care.

## Conclusions

Social media can be implemented as a source of support and guidance for surgical patients, validating physician recommendations and medical information as a whole. Our findings indicate that most Reddit users had positive outlooks on the tonsillectomy procedure, often sharing their personal experiences and seeking validation for symptoms and emotions. Positive outlooks anecdotally increase with time from surgery, while the immediate surgical pain and healing subsides. Few posts involved clinical content, signifying that healthcare providers can best optimize patient satisfaction and care by tailoring to patients’ emotional needs. The prevalence of postoperative questions and the descriptor “pain” among clinical posts suggests that patients turn to Reddit to ask questions and share advice about postoperative pain care regimens. These findings can allow healthcare professionals to better understand individualized care plans, including nontraditional home remedies, and enhance patients’ emotional experiences, minimizing fear and establishing patient-physician trust. In future work, a cumulative analysis of specific support and recommendations within Reddit posts may inform physicians of common advice given online to manage pain. Future research of non-anonymous social media platforms should examine the impact of age and other demographics on sentiment surrounding the tonsillectomy procedure.
